# Supplementary material for: A method for low-coverage single-gamete sequence analysis demonstrates adherence to Mendel’s first law across a large sample of human sperm
Source: eLife. 2022 Dec 7;11:e76383. doi: 10.7554/eLife.76383 (PMC9844984; doi:10.7554/eLife.76383)
Supplement: Transparent reporting form [file elife-76383-transrepform1.pdf]

Westbrook Centre
Milton Road
Cambridge CB4 1YG, UK

P 01223 855340
W elifesciences.org
T @elife

eLife's transparent reporting form

We encourage authors to provide detailed information *within their submission* to facilitate the interpretation and replication of experiments. Authors can upload supporting documentation to indicate the use of appropriate reporting guidelines for health-related research (see EQUATOR Network), life science research (see the BioSharing Information Resource), or the ARRIVE guidelines for reporting work involving animal research. Where applicable, authors should refer to any relevant reporting standards documents in this form.

If you have any questions, please consult our Journal Policies and/or contact us: editorial@elifesciences.org.

Sample-size estimation

- You should state whether an appropriate sample size was computed when the study was being designed
- You should state the statistical method of sample size computation and any required assumptions
- If no explicit power analysis was used, you should describe how you decided what sample (replicate) size (number) to use

Please outline where this information can be found within the submission (e.g., sections or figure legends), or explain why this information doesn't apply to your submission:

To evaluate the statistical power of our transmission distortion (TD) scanning approach, we conducted simulations of various levels of TD (0-20% deviations from Mendelian expectations) across a range of sample sizes of human sperm (Fig. 4, Fig.4-supplemental figure 1), including that of our available data (from Bell et al. 2020 and Leung et al. 2021). From this analysis, we were able to estimate the minimum rate of TD we would be able to observe in our dataset of human sperm (969-3,377 cells from each of 25 donors).

To benchmark rhapsodi's performance with simulated data (Fig. 2, Fig. 2-supplemental figures 1-10, Fig. 3, Fig. 3-supplemental figures 1-2), no explicit power analysis was used to decide on the number of independent technical replicates assessed for each study design. Due to the number of study designs assessed and the practical limitations of computation runtime, we followed the Law and McComas (doi: 10.1016/B978-0-12-386485-7.00003-X) recommendation to estimate performance with 3 replicates.

Because Fig. 4-supplemental figure 2 and Fig 4.-supplemental figure 3 are each used to provide an example of a possible signature of TD due to linkage disequilibrium, a single simulation is displayed and no formal power analysis was performed. This is described in the figure caption and the sections "Application to data from human sperm" (Results) and "Assessing performance with simulation" (Methods).

To build the null distribution for a potential global signal of TD (Fig. 6), 500 independent simulations were run. This number of replicates was determined based on the practical limitations of computation runtime and the need to produce a 2 decimal precision p-value.

eLife Sciences Publications, Ltd is a limited liability non-profit non-stock corporation incorporated in the State of Delaware, USA, with company number 5030732, and is registered in the UK with company number FC030576 and branch number BR015634 at the address Westbrook Centre, Milton Road, Cambridge CB4 1YG.

Replicates

- You should report how often each experiment was performed
- You should include a definition of biological versus technical replication
- The data obtained should be provided and sufficient information should be provided to indicate the number of independent biological and/or technical replicates
- If you encountered any outliers, you should describe how these were handled
- Criteria for exclusion/inclusion of data should be clearly stated
- High-throughput sequence data should be uploaded before submission, with a private link for reviewers provided (these are available from both GEO and ArrayExpress)

Please outline where this information can be found within the submission (e.g., sections or figure legends), or explain why this information doesn't apply to your submission:

The investigation for transmission distortion (TD) was conducted once for each donor (results shown in Fig. 5). Because each sample originates from a unique donor, they are not biological replicates. In quantifying a global signal of TD (Fig. 6), we conducted 500 independent simulations to build a null distribution of allele sharing (stated in Fig. 5 legend). We used the sperm genome data from Bell et al. 2020 and Leung et al. 2021, applied filtering steps (as discussed in Methods section "Genotype filtering to mitigate spurious TD signatures"), and then used all sperm cells remaining (N = 41,189; 969-3,377 cells from each of 25 donors, as stated Abstract and Introduction) in our analysis. No outliers were encountered. Data are accessible as described in "Availability of data and materials."

To benchmark rhapsodi's performance with simulated data, 3 independent replicates were performed for each study design. This is described in the "Assessing performance with simulation" section within the Methods. For those experiments displayed in Fig. 2, Fig. 2-figure supplement 2, and Fig. 2-figure supplements 5-8, each study design is described according to the mean performance. The figure legend of each of these figures states this information. For the other experiments which were used to benchmark rhapsodi's performance with simulated data (displayed in Fig. 3, Fig. 3-figure supplements 1-2, and Fig. 2-figure supplement 3), each of these replicate trials is considered and displayed as independent data points within the overall dataset, as described in the legends of these figures. Simulations to determine window size (displayed in Fig 2.-figure supplement 4) used 1-3 independent replicate simulations for each combination of parameters. This is stated in the Methods section "Automatic phasing window size calculation". To benchmark rhapsodi's computational run time, each combination of parameters was simulated in 3 independent replicates (Fig. 2-supplemental figure 10). This is stated in the Methods section "Benchmarking run time".

To evaluate the statistical power of our TD scanning approach in Fig. 4 and Fig 4.-supplemental figure 1, 1000 independent simulations were performed for each study design. This is stated in the Methods section "Power analysis for detecting TD" and the figure caption. To demonstrate the statistical power of a TD scanning approach in human pedigrees (Fig 4.-supplemental figure 4), 1000 independent simulations were performed for each study design. This is stated in the figure caption.

Simulation replicates (i.e., repeated runs of random simulations) versus biological replicates (i.e., study subjects) are clearly described as such in the text and Methods.

eLife Sciences Publications, Ltd is a limited liability non-profit non-stock corporation incorporated in the State of Delaware, USA, with company number 5030732, and is registered in the UK with company number FC030576 and branch number BR015634 at the address Westbrook Centre, Milton Road, Cambridge CB4 1YG.

Statistical reporting

- Statistical analysis methods should be described and justified
- Raw data should be presented in figures whenever informative to do so (typically when N per group is less than 10)
- For each experiment, you should identify the statistical tests used, exact values of N, definitions of center, methods of multiple test correction, and dispersion and precision measures (e.g., mean, median, SD, SEM, confidence intervals; and, for the major substantive results, a measure of effect size (e.g., Pearson's r, Cohen's d)
- Report exact p-values wherever possible alongside the summary statistics and 95% confidence intervals. These should be reported for all key questions and not only when the p-value is less than 0.05.

Please outline where this information can be found within the submission (e.g., sections or figure legends), or explain why this information doesn't apply to your submission:

(For large datasets, or papers with a very large number of statistical tests, you may upload a single table file with tests, Ns, etc., with reference to sections in the manuscript.)

We used a two-tailed binomial test to investigate transmission distortion (TD) in each donor's set of sperm (as stated in Methods section "Genome-wide scan for TD"). Raw data is not presented. The total number of hypothesis tests as well as the methods used for multiple testing correction are described in the Methods section "Significance threshold for TD scan." Exact p-values for the TD analysis are shown in Fig. 5 and Fig. 6. The power analysis also applied two-sided binomial tests, as depicted with and without multiple testing correction in Fig. 4 and Fig.4-supplemental figure 1 and described in Methods section "Power analysis for detecting TD."

We used a one-tail hypothesis test in quantifying global signatures of TD. This is described in the Methods section "Calculation of global signal of TD." The exact p-value and number of simulations are reported in the caption of Fig. 6B as well as the main text.

For simulation experiments benchmarking rhapsodi's performance, the definitions of center are described in the figure captions (Fig. 2, Fig. 2-figure supplement 2, and Fig. 2-figure supplements 5-8) as well as throughout the Results section "Evaluating performance on simulated data." The definitions of dispersion/precision are also stated in the Results section "Evaluating performance on simulated data," specifically: "(Values are reported as the mean, plus or minus one standard deviation)." In describing the application of rhapsodi to the human sperm genomes, the definitions of center and dispersion/precision are stated in the Results section "Application to data from human sperm," specifically; "(Values are reported as the mean, plus or minus one standard deviation.)"

Group allocation

- Indicate how samples were allocated into experimental groups (in the case of clinical studies, please specify allocation to treatment method); if randomization was used, please also state if restricted randomization was applied
- Indicate if masking was used during group allocation, data collection and/or data analysis

eLife Sciences Publications, Ltd is a limited liability non-profit non-stock corporation incorporated in the State of Delaware, USA, with company number 5030732, and is registered in the UK with company number FC030576 and branch number BR015634 at the address Westbrook Centre, Milton Road, Cambridge CB4 1YG.

Please outline where this information can be found within the submission (e.g., sections or figure legends), or explain why this information doesn't apply to your submission:

Other than the natural grouping of datasets based upon simulated study designs (e.g., number of gametes, number of SNPs, sequencing coverages, recombination rates, etc.) samples were not allocated into experimental groups. Therefore, this information does not apply to our submission.

Additional data files ("source data")

- We encourage you to upload relevant additional data files, such as numerical data that are represented as a graph in a figure, or as a summary table
- Where provided, these should be in the most useful format, and they can be uploaded as "Source data" files linked to a main figure or table
- Include model definition files including the full list of parameters used
- Include code used for data analysis (e.g., R, MatLab)
- Avoid stating that data files are "available upon request"

Please indicate the figures or tables for which source data files have been provided:

Code for all analyses is available on github at https://github.com/mccoy-lab/transmission-distortion/ and model definition files are available at https://github.com/mccoy-lab/rhapsodi (both links included in "Availability of data and material"). The full list of non-constant parameters used in the simulations is provided in the Methods section "Assessing performance with simulation." All other parameters match the defaults in the model definition files.

eLife Sciences Publications, Ltd is a limited liability non-profit non-stock corporation incorporated in the State of Delaware, USA, with company number 5030732, and is registered in the UK with company number FC030576 and branch number BR015634 at the address Westbrook Centre, Milton Road, Cambridge CB4 1YG.
